# Facts from the Small-Pox Hospital at Troy, New York

**Published:** 1881-06-20

**Authors:** Clarkson C. Schuyler

**Affiliations:** Assistant Surgeon Troy Hospital


### FACTS FROM THE SMALL-POX HOSPITAL AT TROY, NEW YORK.

BY CLARKSON C. SCHUYLER, M.D., ASSISTANT SURGEON TROY HOSPITAL.

What I have to offer is merely a resume of a service of four months at the small-pox hospital in Troy. As every phase of the disease was noted, and some unusual phenomena observed, I have thought that this recapitulation might not be uninteresting, inasmuch as the disease still prevails, and some of you, I understand, have it in your midst. While, as will be inferred from what I have said, I do not propose to make this a treatise on small-pox, I would like, however, to call your attention to what are to me some interesting facts in the etiology and symptomatology of the disease of which I took cognizance.

While there is little doubt that the greatest danger from infection in this disease is during the stage of suppuration and desiccation, I am led to believe, from the history of cases noted in families where its members have successively contracted the disease, that there is no stage in which it cannot be communicated; that the breath and exhalations from the body during that part of the period of incubation immediately preceding the stage of invasion can infect the unprotected. These views, while they are contrary to general belief, confirm the observations of Prof. Lomas, who says: "There are well authenticated cases which prove to us that infection may take place during any stage of the disease, even during the period of incubation." I believe, however, that the danger from exposure during the stage of incubation, invasion and the appearance of the eruption, is slight, if the patient be in a large and airy apartment.

It is well known that rarely does an individual suffer from a second attack of the disease. I have seen, I think, three exceptions, there being room for doubt in but one, in which case no evidence of a previous attack could be found upon the body. The history, however, given by the mother of the girl (whose age was 16), seemed to warrant the belief. She said that when about four years of age she contracted the disease from her father; that she was nursed in the room with the father as soon as the disease manifested itself, which was during the period of desiccation. The other cases, both men, aged respectively 36 and 37, were scarred. The former died of the *hemorrhagic* form of variola. It is a fact worthy of note that in each of these three instances the primary attack way previous to puberty. I say worthy of noted, because many believe that if the protective power of vaccination during infancy is ever lost, it is at this period in life.

The precursory signs, excepting, of course, the fever, were in some cases wanting, the pronounced headache and backache, which are as a rule present, having been wholly absent. It was also observed that when the eruption did not appear until the fourth day it was invariably *discrete*. In the discrete form it was often difficult to find umbilicate vesicles at any stage of the disease. (In diagnosis this might be a source of error if small-pox were not prevailing.)

In many instances I had noticed upon the forehead and breast, in this variety, a few aborted vesicles (I refer to cases admitted on the third or fourth day of the disease, or after the appearance of the eruption). This question immediately presented itself: Did these isolated vesicles come in sight, umbilicate and abort previous to the appearance of the general eruption? I was soon able, in two instances at least, to answer this question in the affirmative.

I was called to see a young woman who was thought to be suffering from a severe cold. This was the second day of the attack. She had had the violent prodromic symptoms of variola, and it was immediately suspected that she was suffering from that disease. Upon close inspection three small umbilicated vesicles were found; one upon the forehead, one at the angle of the lower jaw, and one on the neck. Forty-eight hours after the general eruption appeared.

Again, I saw, with a brother physician, an old gentleman, on the morning of the third day of his sickness. He had no marked headache or backache; temperature 103½°, pulse quick, tongue dry, kidneys excreting but little. A few umbilicated vesicles were found upon the forehead, one or two of which were slightly crusted. The eruption made its appearance twenty-four hours later. Whether this is usual I am not prepared to say. I am not aware, however, that it has ever been observed. Bartholow, in his work on "Practice of Medicine," refers to a form of eruption appearing in clusters or patches, which he terms "*corymbic*." But one case having this variety of eruption was seen. Only a single cluster, the size of a silver dollar, appeared upon the face, upon the left cheek. There were a few isolated vesicles, however, at the margin of the hair, on the forehead. A number of patches were present upon the legs and arms, the skin between them being wholly destitute of eruption for a distance, in some places, of at least twelve inches. The vesicles in some of the clusters were coherent, or in immediate contact, without being really confluent.

Death was inevitable, as a rule, in the hemorrhagic variety, or what is popularly known as "black small-pox." Death usually took place before the period of pustulation was reached. In those that died, not only were the vesicles filled with bloody serum, but there were extravasations of blood beneath the cutaneous layers, and hemorrhages from the various orifices of the body. I have seen them die as early as the third day. Two adults recovered, one of which suffered from abscesses for a month after.

Two cases were admitted during pregnancy, five and six months respectively; one hemorrhagic, the other discrete. The former aborted on the fourth day and died on the fifth; the latter made a good recovery, without death to the fœtus.

The pharyngitis in some cases was so severe as to render deglutition impossible. As this usually occurred during salivation, the condition of the patient was most distressing. An authority on small-pox,* speaking of confluent variola, says: "While the eruption may be completely confluent on the face and hands, on other parts of the body it remains distinct, and never becomes confluent except over limited spaces." In two cases the eruption was confluent on every part of the body—one, a young woman aged 20, the other a girl of 15. The former died on the tenth day of the disease, the latter on the ninth.

The performance of laryngotomy in one case apparently suffering from œdema of glottis was seriously thought of; the patient recovered, however. No diphtheritic membrane was observed in these cases, nor in the hemorrhagic form, as is spoken of by some authorities. No uncontrollable vomiting or diarrhœa was met with. These concomitants of the period of invasion were rarely seen thereafter.

And now, as to treatment. The question naturally presents itself, Do we possess any power to arrest the development or mitigate the severity of this disease? As to the development, I answer, no; excepting, of course, vaccination immediately after exposure, which, if successful, will insure a modified form of the disease. There are certain stages, the severity of which may be mitigated, especially that of invasion, with restlessness and high temperature, when aconite, given hourly, has a most happy effect. Bitartrate of potash, in the proportion of an ounce to a pint of water, taken *ad libitum*, was a grateful drink. It seemed to limit the inflammation of the skin and hasten desiccation. At any rate, those taking it "cleaned off quicker."

In severe cases you are compelled to resort to the use of stimulants, heroically, too, sometimes. Many a life has, I believe, been saved by the judicious use of stimulants at the period of suppuration, when the patient is found with a dry tongue, quick pulse, blue lips, and, sometimes, active delirium—a condition very like the last stages of typhoid. Frequent sponging during the development of the eruption was found to be very grateful.

Nothing I found gave so much comfort during the stage of desiccation as a warm bath, the patient frequently begging to have it repeated. As an instance showing the benefit derived from the baths, I may mention the case of a young man who was brought to the hospital on the sixteenth day of the disease. He was a most frightful object as he lay there before me, with hardly the semblance of a human being, unconscious and in a muttering delirium. As a *dernier resort*, a warm bath and stimulants were ordered. In three-quarters of an hour he was removed, placed in bed and freely oiled. Within one hour he was conscious and able to tell of the suffering and neglect he had undergone previous to admission. Nothing, I believe, can be relied upon when we deal with the hemorrhagic variety; although tinct. chloride iron with turpentine seemed to be given with good effect in the two cases that recovered.

Vomiting was invariably controlled with subnitrate of bismuth, taken dry, in 10 or 15 grain doses. What was done to prevent pitting? you are no doubt ready to ask. Well, nothing, because I believe nothing can be done. The pitting depends entirely upon the depth of the slough under each pustule. If the infiltration of the pus cells into the vesicles takes place without extension of inflammation into the cellular tissue beneath, then there will be no pitting; but if it does extend into the deeper tissues and a slough is the result, then pitting is inevitable.

That some are scarred more than others is a natural course of the disease.

The whole number of cases treated was 216. I have a record of 199. As to those who died previous to my keeping a complete record, a memorandum, as to age, vaccination and variety of disease, was made.

A perusal of the following table will be of interest:

| | Number. | Varioloid. | Discrete. | Hemorrhagic. | Confluent. | Corymbic. | Died. |
|---|---|---|---|---|---|---|---|
| | | Variety | | | | | |
| Not vaccinated, . . . . . . . | 105 | 7 | 50 | 12 | 35 | 1 | 33 |
| Vaccinated after exposure, . . . | 2 | 2 | — | — | — | — | — |
| Vaccinated in infancy, . . . . . | 64 | 21 | 24 | 8 | 11 | — | 10 |
| Vaccinated, . . . . . . . . . | 17 | 11 | 5 | — | 1 | — | 2 |
| Inoculated, . . . . . . . . . . | 8 | 3 | 2 | 2 | — | — | 1 |
| Had small-pox, . . . . . . . . | 3 | — | 2 | 1 | — | — | 1 |

| Ages. | Number. | Deaths. | Percentage of death. |
|---|---|---|---|
| Infants, . . . . | 11 | 5 | 45.5 |
| 1 to 10 years, . . . | 68 | 15 | 22.0 |
| 10 to 20 years, . . . | 44 | 12 | 27.2 |
| 20 to 30 years, . . . | 46 | 8 | 17.4 |
| 30 to 40 years, . . . | 18 | 5 | 27.7 |
| 40 to 50 years, . . . | 8 | 1 | 12.5 |
| 50 to 60 years, . . . | 4 | 1 | 25.0 |
| Total, . . . | 199 | 47 | |

It will be observed that the mortality was about 22 per cent., which we think is a very good showing, taking into consideration the type of the disease that has prevailed. Probably at no time in the history of small-pox in Troy has the disease been so malignant. And, too, the number of cases admitted when the disease was far advanced necessarily increased the ratio of mortality, premising that the hospital care and treatment were superior to that received at home. In the "vaccinated" all children were carefully examined as to scar. I mention this, as it will be seen, from the table, eleven had varioloid, five discrete, and one confluent small-pox. Believing, as I do, that vaccination is an absolute protection against small-pox (not varioloid), I am positive that those having the discrete and confluent forms were unprotected.

Not a single case having a recent vaccination was admitted during my service. One child having a recent vaccination (within two weeks) was admitted, with four others of the same family having the disease, and remained in the hospital for two months without contracting the disease.

The greatest mortality, it will be observed, was among infants, 45 per cent. dying. The lowest rate was between the ages of forty and fifty, only 12 per cent. dying.

I cannot close this paper without saying a word in commendation of the heroic fortitude, patient devotion and Christian graces of the Sisters of Charity, who voluntarily connected themselves with the institution. The success of the treatment of patients therein and the low mortality among them are due, in great measure, to the watchful care and attention of these Sisters, who have shown themselves to be real ministering angels to the afflicted.—*Med. and Surg. Reporter.*

